# Ultra-high resolution HLA genotyping and allele discovery by highly multiplexed cDNA amplicon pyrosequencing

**DOI:** 10.1186/1471-2164-13-378

**Published:** 2012-08-06

**Authors:** Simon M Lank, Brittney A Golbach, Hannah M Creager, Roger W Wiseman, Derin B Keskin, Ellis L Reinherz, Vladimir Brusic, David H O’Connor

**Affiliations:** 1 Wisconsin National Primate Research Center, University of Wisconsin-Madison, Madison, WI, USA; 2 Cancer Vaccine Center, DanaFarber Cancer Institute, Boston, MA, USA; 3 Department of Medicine, Harvard Medical School, Boston, MA, USA; 4 Department of Pathology and Laboratory Medicine, University of Wisconsin-Madison, Madison, WI, USA

**Keywords:** HLA, Genotyping, Roche/454, Pyrosequencing, Galaxy, Tissue typing, Cellular immunity, Multiplexing

## Abstract

**Background:**

High-resolution HLA genotyping is a critical diagnostic and research assay. Current methods rarely achieve unambiguous high-resolution typing without making population-specific frequency inferences due to a lack of locus coverage and difficulty in exon-phase matching. Achieving high-resolution typing is also becoming more challenging with traditional methods as the database of known HLA alleles increases.

**Results:**

We designed a cDNA amplicon-based pyrosequencing method to capture 94% of the HLA class I open-reading-frame with only two amplicons per sample, and an analogous method for class II HLA genes, with a primary focus on sequencing the DRB loci. We present a novel Galaxy server-based analysis workflow for determining genotype. During assay validation, we performed two GS Junior sequencing runs to determine the accuracy of the HLA class I amplicons and DRB amplicon at different levels of multiplexing. When 116 amplicons were multiplexed, we unambiguously resolved 99%of class I alleles to four- or six-digit resolution, as well as 100% unambiguous DRB calls. The second experiment, with 271 multiplexed amplicons, missed some alleles, but generated high-resolution, concordant typing for 93% of class I alleles, and 96% for DRB1 alleles. In a third, preliminary experiment we attempted to sequence novel amplicons for other class II loci with mixed success.

**Conclusions:**

The presented assay is higher-throughput and higher-resolution than existing HLA genotyping methods, and suitable for allele discovery or large cohort sampling. The validated class I and DRB primers successfully generated unambiguously high-resolution genotypes, while further work is needed to validate additional class II genotyping amplicons.

## Background

The human leukocyte antigen (HLA) complex on the short arm of chromosome 6 encodes the most polymorphic genes in the human genome, and is extensively studied due to the central role its associated proteins play in the etiology of infectious diseases, autoimmune disorders, and organ transplantation outcomes [1-5]. The “classical” HLA class I and II gene products are involved in endogenous and exogenous antigen presentation respectively, mediating adaptive immune responses to pathogens and tissue transplants. As sequencing methods of characterizing human genetic diversity have proliferated, the number of known classical HLA alleles has exploded, with over 6,800 distinct variants described to date, 1,633 of which were identified in 2010 alone [6]. The majority of observed allele variants occur within the class I genes HLA-A, -B and -C, as well as the class II DRB genes. These highly polymorphic loci are the most commonly studied for their differential effects on directing adaptive immunity [7,8]. The rapid proliferation of reference alleles in the curated IMGT database demonstrates that coding HLA polymorphism is vast, with certain serologically distinct families containing many hundreds of individual alleles. For instance, in May of 2011, four new HLA-A*02 alleles were described [9] with a total of 334 protein-distinct alleles of this lineage described to date.

Non-synonymous allelic variants of HLA gene products can bind distinct antigenic peptides [10,11], be subject to differential regulation [12,13], and have varied interactions with T-Cell Receptors [14], Killer Immunoglobulin-like Receptors[15] and viral proteins [16]. The direct link between HLA polymorphism and various diseases is a subject of intensive investigation, with hundreds of published studies every year. High-resolution HLA typing (to the specific protein, rather than the serological group level) can elucidate the differential consequences of small protein changes within an allele family, such as the alleles B*57:02 and B*57:03, which can differ by a single nucleotide yet lead to differential outcomes during HIV infection [17]. The ability to type individuals at true high-resolution may uncover further links between individual alleles and disease.

Traditional HLA typing methods are losing their effectiveness at accurately predicting high-resolution (four-digit) genotype due to the large number of highly similar alleles. Molecular sequence specific PCR (SSP) and sequence specific oligonucleotide hybridization (SSO) methods use unique sequence signatures to detect the presence or absence of an allele; however, with more alleles being described every month, many with only one novel single nucleotide polymorphism (SNP), it is impossible for SSP panels to rule out the presence of uncommon but similar alleles. For higher accuracy, these methods rely on an ever-greater number of reactions to distinguish potentially related alleles, or use complementary methods to resolve ambiguities [18]. More sophisticated (and expensive) sequence-based methods improve resolution; however, even “gold-standard” commercial kits fail to produce the entire coding sequence of individually typed alleles. Allele ambiguity is an increasing problem as many new alleles have been described with SNP variants outside of the traditional exons examined by sequence-based typing (SBT). This has led to the proliferation of methods for imputing four-digit genotype from limited sequencing data by utilizing known allele frequencies in various populations [19-21].

Traditional typing methods thus cannot always distinguish between highly similar alleles that may differ by a single SNP within the amplification region (SSP and SSO) or outside of it (SBT), and using allele frequency to inform genotyping can lead to results biased against rare variants. For the highly polymorphic HLA genes, SBT (Sanger- or Roche/454-based) usually only interrogates the class I exons 2 and 3 (or 2, 3 and 4), and exon 2 for class II alleles [22,23]. While many typing kits and software may appear to generate unambiguous results with their methods, they do not consider alleles newly added to recent IMGT database versions. Single SNP differences between related alleles in the non-assayed exons could have potential regulatory or immunologic consequences, although few studies have examined such correlations due to the difficulty of sequencing the additional regions. In fact, when considering the current database, many common HLA class I and class II alleles (particularly the highly polymorphic DRB locus) remain four-digit ambiguous with exon 2,3,4 typing (class I) and exon 2 typing (DRB) [Table 1 and (Additional file 1: Table 1). To date, full allele sequencing and genotyping are rarely employed due to the associated cost of multi-exon amplification and sequencing, and difficulty of determining exon phase, particularly for the small, less polymorphic 3’ exons. 

**Table 1:** Four-digit ambiguous alleles with standard SBT methods and cSBT

**Class I exon 2, 3, 4 ambiguous to 4-digit (high freq)**	
*Allele*	*dbMHC freq*
C*07:02:01/07:50	13.20%
C*04:01/04:09 N	11.40%
C*07:01/07:06/07:18	8.20%
C*08:01/08:22	4.90%
B*07:02:01/07:61	4.80%
B*35:01:01/35:42:01	4.50%
16 others	variable
DRB Exon 2 Ambiguous to 4-digit (high freq)	
*Allele*	*dbMHC freq*
DRB1*11:01/11:100	6.60%
DRB1*14:01:01/14:54	4.10%
6 others	variable
All cSBT ambiguous to 4-digit	
*Allele*	*dbMHC freq*
A*74:01/74:02	0.90%
C*07:04/07:11	1.70%
B*27:05/27:13	1.50%
DRB1*12:01:01/12:10	1.80%

“Gold Standard” Sanger sequence-based genotyping usually amplifies a minimal number of exons per allele, resulting in a large number of ambiguous allele calls. A non-inclusive list of commonly observed alleles with four-digit ambiguity using exon-based typing methods is shown for class I (exon 2, 3, & 4) and DRB (exon 2) alleles. Also included is a list of all four allele combinations predicted to be four-digit ambiguous using the expanded cSBT method.

Recently, we described a novel HLA class I genotyping method (cSBT), utilizing a PCR amplicon that universally amplifies a 581 bp diagnostic template from cDNA, encompassing all of exon 3 plus portions of exons 2 and 4 [24]. This method allows unprecedented throughput for class I HLA typing (through the use of a single universal PCR amplification) but lacks the ability to fully distinguish class I exons 2 and 3 (the minimal requirements for novel allele discovery), producing statically ambiguous genotyping results.

The primary aim of this study was to expanded the area of sequence coverage using novel primer binding sites in exons 1 and 7 of the HLA class I open reading frame, pan-locus DRB primers, and preliminary use of universal primers for the other class II loci (DRA, DPA, DPB, DQA, and DQB) to unambiguously identify HLA genotypes in large patient cohorts with unprecedented resolution and throughput without relying on the use of population-specific allele frequency inferences [20,23]. We also designed an open-source analysis workflow for rapidly imputing high-resolution genotype. In the process, several novel HLA alleles were identified and validated with direct PCR Sanger sequencing. Thus, the improved cSBT method allows rapid screening of large cohorts for potential novel allele characterization. This general approach will also be adaptable to improving second- and third-generation sequencing technologies that may allow longer amplicons to be completely sequenced [25,26].

## Results

### HLA Class I primer design

**Figure 1:**
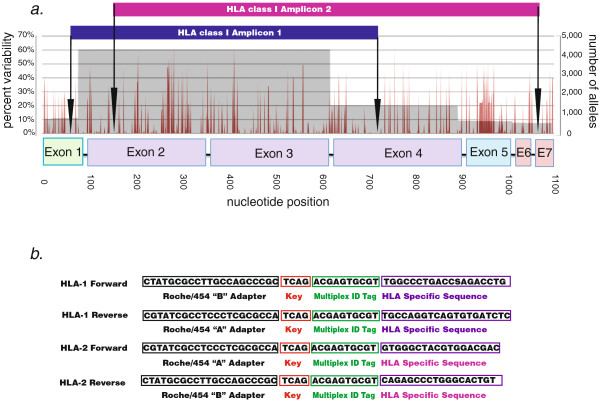
**Positioning and sequence of HLA class I universal amplification primers.** An alignment of >4,000 known HLA class I alleles produced a variability profile. (**a**) The depth of coverage (i.e. the number of alleles at each position in the alignment) is shown in grey, along with the percentage of nucleotide sequence variability (red) and the positioning of the two genotyping amplicons. (**b**) The sequence and composition of universal amplification primers for amplicon 1 and 2. Note that for amplicon 1 the “A” and “B” adapters were swapped to avoid a primer amplification problem.

Alignment of all described HLA class I alleles revealed several conserved sequence regions suitable for designing pan-locus amplification primers. In addition to previously described primer sites for a 581 bp diagnostic cSBT amplicon [24], other sites in exons 1 and 7 of the class I open reading frame were used to produce two overlapping genotyping amplicons (HLA-1 andHLA-2) of size 676 bp and 925 bp covering nucleotides 50–725 and 145–1069 respectively (Figure 1). We predicted the resolution of the expanded assay *in silico* by aligning 3,665 class I alleles over the area of sequence coverage, and found only three 4-digit allele pairs that are ambiguous as of the version 3.4 IMGT database Table 1. Since theamplicons capture over 90% of the coding sequence, and the majority of new alleles are described only from exon 2,3 or 2,3,4 sequence only we do not expect that the number of ambiguous allele possibilities will dramatically increase with newer database versions. 

### HLA Class II primer design

**Figure 2:**
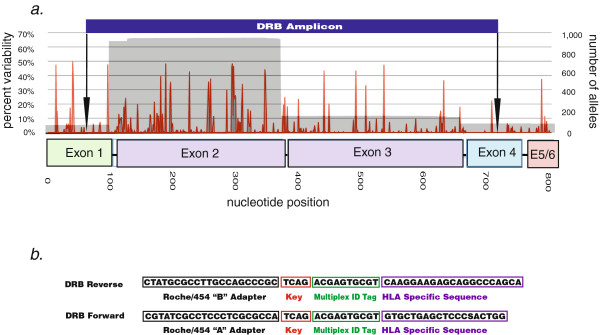
**Positioning and sequence of HLA DRB universal amplification primers.** An alignment of all known HLA DRB alleles produced a variability profile. (**a**) The depth of coverage (i.e. the number of alleles at each position in the alignment) is shown in grey, along with the percentage of nucleotide sequence variability (red) and the position of the DRB genotyping amplicon. (**b**) The sequence and composition of universal amplification primers for the DRB amplicon.

We applied the same method to all HLA class II loci to identify universal amplification primers. For the class II DRB locus (the most polymorphic class II gene) primers at positions 61–79 and 707–727 generated a 667 bp amplicon (Figure 2) that is universally conserved, except for auxiliary DRB3 alleles, which contain a single nucleotide mismatch in the 5’ primer (C74G). Similar to the class I alignment, *in silico* analysis of 966 DRB alleles identified only a single 4-digit ambiguous allele combination Table 1. Using identical methods we also identified preliminary universal primers for the other class II loci DRA, DPA, DPB, DQA and DQB (Additional file 1: Table 2).

### Library preparation and pyrosequencing

We generated cDNA from total cellular RNA, and PCR-amplified the three primary diagnostic HLA amplicons described above (HLA-1, HLA-2 and DRB) from 98 separate samples, including 40 International Histocompatibility Working Group reference cell lines http://ihwg.org. For a limited set of four reference samples we also generated amplicons from the additional class II loci. The quality and purity of the DNA amplicon sequencing library is important to ensure successful sequencing [27], so we performed multiple solid-phase reversible immobilization (SPRI) clean-ups on our samples to remove excess primer-dimer and shorter PCR products. We used 96 distinct multiplex identifier (MID)-tagged primer pairs for each amplicon to generate sequencing libraries Additional file 1: Table S3.

The presence of differently sized amplicons in the sequencing library is an important consideration. The final amplicon sizes of the primary products (including MIDs and adapter sequences) were 746 bp (HLA-1), 995 bp (HLA-2), and 737 bp (DRB Amplicon). Although HLA-2 is longer than typically recommended for Roche/454 pyrosequencing, the use of an augmented pooling strategy accounting for the differential sizes of amplicons (longer products at a higher molar ratio than the shorter ones) produced normalized sequence data. This was necessary since the Roche/454 emPCR process preferentially amplifies shorter PCR products.

We attempted to sequence these amplicons over the course of two Roche/454 GS Junior sequencing runs to test the performance of the three amplicons and the multiplexing limit of the Roche/454 GS Junior for our assay respectively. Both sequencing runs performed well, yielding a variable number of sequence reads depending on multiplexing level, as well as the filter-pass performance of each amplicon at the augmented pooling ratios Table 2 and Additional file 1: Table S4. A third sequencing experiment of the additional class II amplicons generated a large number of reads per amplicon due to the low multiplexing ratio Additional file 1: Table S5.

**Table 2:** Read metrics for both sequencing runs described

	**Run 1**	**Run 2**
Raw Wells	194,989	220,452
Keypass Wells	193,002	218,741
Filterpass Wells	74,101	77,977
Ave Length (st. dev)	482 (108)	487 (91)
Samples Multiplexed	52	92
Amplicons Multiplexed	116	271
Reads per Sample (st. dev)	1,319 (520)	825 (504)
Amp 1 Reads per Sample (st.dev)	542 (167)	386 (142)
Amp 2 Reads per Sample (st. dev)	592 (272)	352 (247)
DRB Reads (st. dev)	803 (378)	92 (79)

The number of raw, keypass, and filterpass reads per run are shown, along with the number of samples multiplexed, and the average read number for each amplicon included in the pool.

### Analysis design and implementation overview

**Figure 3:**
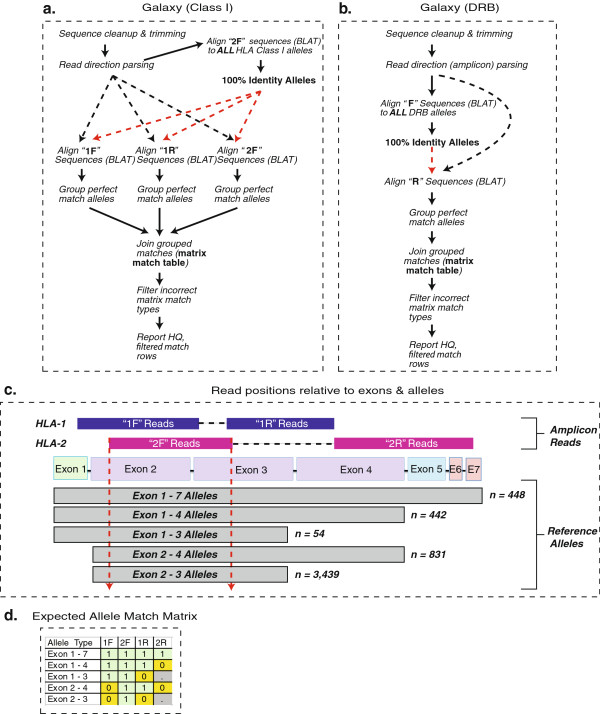
**Overview of mapping pipeline using Galaxy server, and generation of an expected allele match matrix.** Our Galaxy-based mapping pipeline is shown for both class I (**a**) and DRB (**b**) sequence reads. The parsed “2 F” reads are used to limit the subset of subsequent alleles for matching since all named class I alleles have 2 F sequences as shown (**c**). The expected allele match matrix is shown for class I alleles (**d**). Type 1 matches are full, 100% identity matches along the entire read length. Type 0 are partial matches, where the read is longer than the reference allele (and contains novel sequence). Null matches (.) indicate no expected read match based on the lack of a reference allele sequence.

Traditional, genomic-sequence-based HLA analysis pipelines and software tools are inadequate forcDNA amplicon sequencing data sets [23,28]. To facilitate rapid analysis of each de-multiplexed data set we designed a standardized workflow, using Galaxy server [29] to map individual reads to the set of reference alleles (3,665 class I, 996 DRB) using the BLAT alignment tool [30]. An overview schematic of the mapping strategy Figure 3 a and b as well as the specific Galaxy workflow are available. The workflow can be ported to any Galaxy instance with the necessary tools installed, including UNIX tools (http://hannonlab.cshl.edu/galaxy_unix_tools/index.html) and a custom Perl tool Additional file 2: File 1. 

The different length of HLA reference alleles relative to the sequence positions necessitates an iterative approach to determining genotypes. To improve data regularity and quality, all reads were subjected to stringent length filtering and trimming (between 300 and 350 bp trimmed reads, depending on the amplicon), which resulted in the loss of 25% to 60% of total filterpass sequences. The remaining normalized sequence reads were mapped at 100% identity to reference alleles, and the pattern and number of sequence matches for each individual was evaluated to deduce genotype.

For samples that sequenced or amplified at lower numbers, and for our second highly multiplexed experiment, we employed a less stringent, modified pipeline that allowed allele-specific read groups of any number. These calls are noted in orange on the genotyping tables and are of intermediate confidence due to the limited number of sequencing reads.

### Class I genotyping analysis

For class I genotyping, allele mapping began with the HLA-2 forward reads (2 F) to determine the constellation of potential alleles present in an individual Figure 3c. This was possible since trimmed 2 F reads encompass only part of exons 2 and 3, and all named alleles have complete exon 2 and 3 sequences. Perfectly matched 2 F reads thus dictated the reference set for subsequent matches, reducing the number of alleles evaluated for subsequent mapping from several thousand to several hundred and dramatically decreasing computation time for subsequent BLAT mapping.

Using this restricted subset of reference alleles, the other trimmed read groups were evaluated for identity. Based on the positioning of these reads relative to the reference, we established match conditions for each potential allele. These conditions included fully matched reads (condition #1), partially matching reads where the query read contained nucleotide positions not known for the reference, such as exon 1 sequence for exon 2/3-only alleles (condition #0), and unmatched reads (condition null). For alleles of different lengths, a distinct matrix of read match conditions is expected Figure 3d. The matrix is generated by performing inner joins of the 2 F dataset to the 1 F and 1R matches, and an outer join with the 2R matches to account for short reference alleles with null 2R match conditions.

### Class II genotyping analysis

For class II data, the same length and quality filtering were applied, and similar issues arose when mapping the forward and reverse reads, as many alleles are known only from exon 2 sequence. In this case forward reads are used as the key to limit the potentially matching allele set, considering matches with extension sequences at both the 5’ and 3’ end valid due to the positioning of the reads relative to exon 2. Reverse reads are then evaluated against a reference subset of only forward matching alleles. A condition matrix, similar to the one used for class I reads, is established. However, due to the prevalence of exon-2-only reference alleles, an additional condition is added for forward reads when the query reads contain additional 5’ *and* 3’ sequence relative to the reference.

### Class I genotyping results

**Figure 4:**
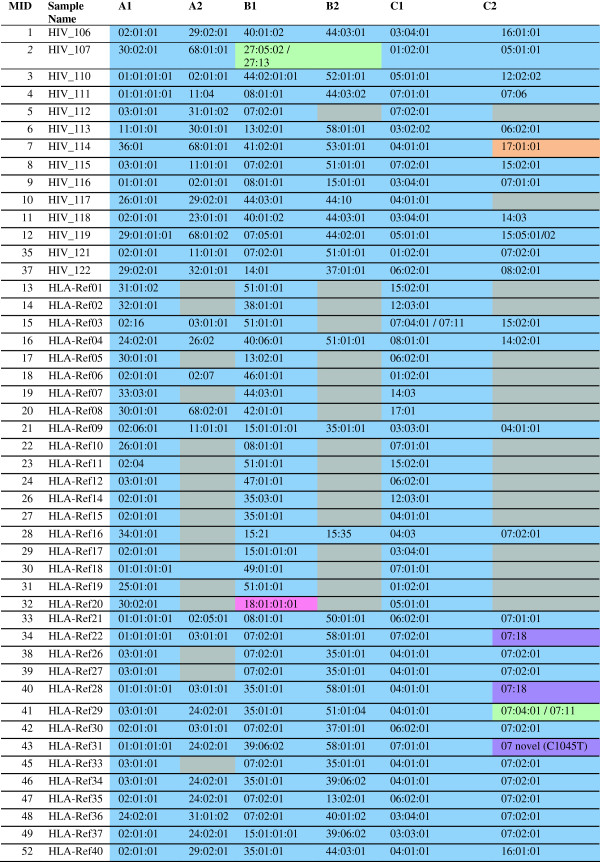
**The HLA class I allele calls from the first genotyping experiment, designed to test the use of the two-tiled class I amplicons, are shown.** Calls in blue were completely unambiguous to the allele level. Calls in green were four-digit ambiguous based on distinguishing SNPs outside the amplified region. The call in orange was only recovered using the modified, lower-stringency analysis pipeline, while the call in pink was expected but not observed for both amplicons. Calls in purple were examples of reference typing undergoing correction, or the detection of a putative novel allele.

The first genotyping experiment produced high-resolution class I allele calls that were 99% concordant to four- or six-digit resolution with previous typing Figure 4. The majority of alleles were typed to six-digit resolution (209 of 224 calls), and most of the remaining four-digit high-resolution calls did not have any associated alleles with six-digit distinctions, and are still considered to be allele-specific matches. One allele (C*17:01:01 from HIV_114) was only detected with the lower stringency, modified analysis pipeline described above. Another allele (B*18:01:01:01 from HLA-Ref20) did not amplify in amplicon 2 due to a single bp forward primer mismatch, accounting for the 1% lack of concordance with previous typing data. In this case, the allele was detected in HLA-1 sequences, but was missed with the analysis pipeline due to the lack of HLA-2 matches. A small number of samples failed to amplify due to sample preparation issues and are not included in the results [data not shown].

Our attempt to push the assay to 92 samples (271 amplicons, both class I and DRB) in a single GS Junior run for the second experiment missed many alleles (not seen, rather than incorrectly called) without using the modified, lower-confidence pipeline. Still, at this multiplexing level our assay yielded high-resolution, accurate genotyping for 93% of expected class I alleles (Additional file 1: Table S6).

Our sequence-based results overturned several incorrect typings in the reference samples that were subsequently confirmed with traditional typing methods. With the additional exon coverage in this experiment, we were able to demonstrate deficiencies in the original SSOP reference cell line typing. This included correcting the original HLA-C*07:01 calls for samples HLA-Ref22 andHLA-Ref28 to HLA-C*07:18. The distinguishing SNP between these alleles occurs at nucleotide 1043, and was not assayed with either previous SSP or SBT methods. We also identified a putative novel HLA-C*07 allele in sample HLA-Ref31.

### Class II genotyping results

**Figure 5:**
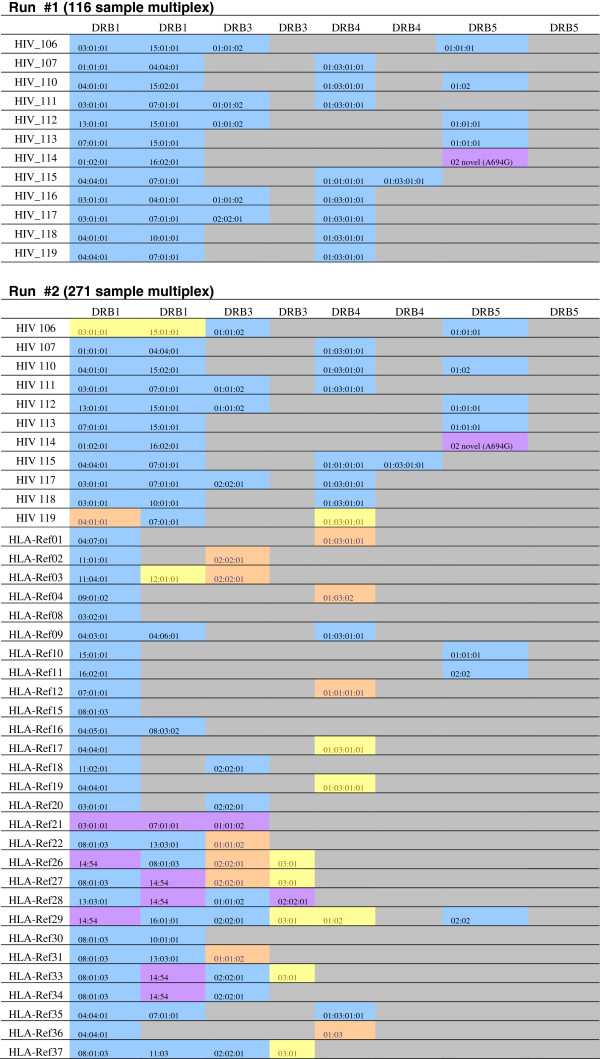
**The resolution and accuracy of the DRB genotyping amplicon in both experiments for HIV patient samples and known reference samples.** Calls in blue were completely unambiguously detected. Calls in orange were detected using the modified, lower-stringency Galaxy pipeline, calls in purple were novel or corrected reference alleles. Calls in yellow were observed in some, but not all of the read directions (typically missed in the 2R read direction) and cannot be considered completely assayable at the indicated multiplexing level. The full DRB genotyping results (including non-reference samples) for the second experiment can be found in Additional file 1: Table S5.

The first experiment applied the DRB amplicon to unknown patient samples as part of a separate study, rather than the HLA reference samples. However, we identified two DRB1 unambiguous, high-resolution allele calls for all 12 samples as expected (Figure 5), as well as a number of DRB3, 4, and 5 high-resolution calls. In the second, highly-multiplexed experiment (which included class I and DRB amplicons for each sample) expected DRB alleles were occasionally missed (similar to the class I results). However, the accuracy of the DRB calls with the lower confidence pipeline resulted in 96% accuracy for DRB1 alleles, and 84% for additional DRB loci alleles, in addition to identifying three putative novel alleles (Additional file 1: Table S7). Considering this data, as well as DRB allele calls in the second experiment, which used the reference samples, the DRB amplicon appears to be successful at establishing correct DRB1 genotypes and of undetermined efficacy for other DRB locus calls at the higher multiplexing levels.

In the third experiment of additional class II loci amplicons we identified alleles from each locus sequenced for all four samples Additional file 1: Table S8. Comparison of this data with the reference class II typing previous done on the samples by SSOP was 72% concordant (13 of 18 alleles matching). However, in several cases our results were discordant with original typing, either identifying different variants of the same serological group, or identifying alleles not seen with SSOP typing. It is unclear whether these differences result from our analysis methods or further deficiencies in the SSOP reference typing, as was the case for several class I alleles in the other experiments.

### Novel allele characterization

As a demonstration of the ability of the assay to detect novel alleles, we chose one of the putative novel sequences (Additional file 3: File 2) identified in sample KTS02 and confirmed its sequence using allele-specific PCR and Sanger sequencing. All alleles present in KTS02 were interrogated, and primers specific for the putative novel allele were identified. Following primary PCR, six allele-specific primers were used to Sanger sequence this product (Additional file 1: Table S9). Analysis of the resultant data confirmed the novel SNP (T587C), resulting in a proline to leucine substitution. This sequence was submitted for approval and the name HLA-C*08:56 was officially assigned by the WHO Nomenclature Committee in October 2011. This follows the agreed policy that, subject to the conditions stated in the most recent Nomenclature Report that names will be assigned to new sequences as they are identified [31]. Lists of such new names will be published in the following WHO Nomenclature Report.

## Discussion

We present an improved version of a previously described cSBT assay with dramatic improvements in resolution through the use of additional universal primer binding sites in the HLA class I open reading frame, the addition of a universal DRB amplicon, and preliminary data from additional class II loci amplicons. Our results demonstrate a scalable technique for use on either the Roche/454 GS Junior or the FLX platform with Titanium chemistry.

The use of a cDNA template, combined with clonal second-generation pyrosequencing, allows comprehensive genotyping from only two PCR amplifications for all HLA class I loci, instead of the nine or more exon- and locus-specific amplifications required for genomic DNA template methods (both Sanger and Roche/454-based). Other amplicons, such as those presented for DRB and other class II loci, can be used to achieve high-resolution genotyping as needed. This updated method allows for dozens or hundreds of patient samples to be HLA class I (and DRB or other loci) genotyped to true high-resolution (allele-specific) simultaneously using fewer amplicons per sample, at a low per-sample cost for both large scale screening projects (1,000 s of samples) using a GS-FLX instrument, or smaller (dozens to hundreds of samples) on a GS Junior instrument. Per-sample cost varies depending on the level of multiplexing and other factors, but can be estimated at ~ $70 - $100 for a 48 to 96 sample multiplexed experiment on a GS Junior instrument (Additional file 1: Table S10). Our results also demonstrate that cSBT has higher throughput and higher resolution than existing methods, and does not rely on population-specific allele frequency inferences.

Higher throughput than reported here (>92 samples typed at a time) is possible for the entire pre-emPCR process in a lab setting utilizing liquid handling robotics for RNA extraction, cDNA synthesis, PCR amplification, SPRI purification, quantification, and pooling, similar to the methods employed by Erlich et al. [23]. Despite the need to generate 288 amplicons per 96 samples typed with the cSBT method for class I and DRB loci, this is a dramatic reduction over the >960 amplicons needed to perform comparably accurate typing from genomic DNA starting material using locus- and exon-specific amplification primers [22,23,28].

An important caveat to the presented data is the different levels of multiplexing used in the first two genotyping experiments. This limited data is insufficient to establish an “ideal” multiplexing level for cSBT. The first GS Junior sequencing run, which multiplexed 116 amplicons (equivalent to 58 class I samples, 39 class I + DRB, or 15 class I + all class II loci) was highly accurate, and is the current recommended multiplexing level. Investigators who consistently recover abundant data may wish to increase the multiplexing level nearer to the second sequencing run (271 amplicons multiplexed), which, in the presented data, performed nearly as well as the lower multiplexed run when lowering sequence number requirements.

Another consideration is the use of and availability of high-quality RNA, which is necessary for cSBT. Traditionally, HLA typing facilities have collected DNA for use with established assays. DNA has the advantage of being easier to collect and store, whereas RNA requires more robust preservation and extraction methods. The limited access to RNA may not make cSBT ideal for all situations, particularly clinical settings that only have access to poor-quality starting material. In such cases a genomic DNA-based method may be more appropriate [22][23]. Current studies, underway with collaborators, are evaluating the use of saliva, buccal swabs, or tissue biopsies as RNA starting material. Initial results with saliva starting material have led to successful amplicon generation. However, class II genotyping likely requires the use of whole blood or PBMC as a starting material, since class II transcripts are unlikely to be expressed in other tissues.

This general approach will be adaptable to improving second- and third- generation sequencing techniques that may allow even longer amplicons to be completely sequenced [25,26]. As longer read-length clonal sequencing technologies become available (such as GS-FLX + from Roche/454 or SMRT sequencing from Pacific Biosciences), it should be possible to use the exon 1 and exon 7 primer sites described here to amplify a single PCR product, instead of the two tiled amplicons that are required currently. We have already successfully amplified and sequenced this ~1 kb amplicon with Roche/454 Titanium sequencing, although the high-quality read lengths were insufficient to provide complete coverage (data not shown). However, the use of a single amplicon increases the likelihood that a subset of alleles with primer incompatibilities could be missed, since a two-tiled amplicon approach produces redundant data with two separate amplifications. Using two class I amplicons acts as an important control to ensure that any allele with primer incompatibilities for a given amplicon is detected with the other, such as the detection of HLA-B*18:01:01:01 in sample HLA-Ref20 despite a primer mismatch for one of the amplicons. In our highly-multiplexed experiment, the loss of one amplicon read direction was more common, even for non-mismatched alleles, and this impacted our ability to distinguish allele resolution, though we were still able to detect signatures of alleles missing from a single read group. The use of a second class I amplicon also facilitates analysis of apparently homozygous loci, which can be artifacts of poorly amplified or potentially novel alleles. It should be noted that although the sequencing platform and read length may change, the cSBT primers and much of the analysis methods presented here would be applicable to any cDNA-based assay.

Methods with more limited coverage than cSBT can lead to incorrect genotype associations. For instance, in our reference panel we were able to determine that two allele calls originally labeled as HLA-C*07:01 were in fact HLA-C*07:18, which encodes a distinct protein. This underscores the power of our approach, which does not assume ambiguously amplified sequences to be the most likely allele in a given population. Additionally, our genotyping results were usually accurate to six-digit resolution, an unprecedented achievement for a high-throughput HLA genotyping assay. While it is currently unknown whether synonymous coding six-digit variants have differential effects on allele expression or disease correlations, it should now be possible to examine this question.

Our preliminary investigations into typing other class II loci have show that the presented primers generate sequencable products, which map to known alleles. However, more work is needed to validate these amplicons properly as we have not determined the cause of observed differences between reference SSOP typing and the presented cSBT typing. We provide this data, and the primers used, so that investigators can carry out further work on these loci if desired.

Two other potential applications of expanded cSBT are novel allele discovery and extending the reference sequence length of known alleles. In the first case, we have shown that cSBT can effectively screen large cohorts for novel alleles, which can then be characterized with traditional and accepted sequencing methods. In the second application, the majority of named HLA class I alleles are known only from exon 2 and 3 sequence (3,439 alleles). Thus, if cSBT was applied to samples with these rare alleles, exons 4, 5, and 6 and parts of exons 1 and 7 could be quickly deduced to improve the length and quality of the IMGT reference database.

## Conclusions

The rapidly expanding number of described classical HLA alleles is undermining the utility of previously unambiguously high-resolution typing assays to provide unambiguous results. The cSBT method presented here offers one solution to overcome the difficulties associated with the ever-expanding HLA allele library: sequence a larger fraction of the open reading frames, and use cDNA starting material to eliminate the problems of exon-phase matching. Combining this with clonal, long-read-length Roche/454 pyrosequencing has produced the ability to unambiguously resolve alleles for large cohorts at a fraction of the cost and time of previous methods. This method also does not rely on population-specific allele frequency inferences, and only uses the a priori knowledge of the conserved primer binding sites to amplify both known and novel alleles. The modified cSBT method for high-throughput, high-resolution HLA typing should accelerate studies involving large cohort genotyping, and allow a deeper survey of worldwide HLA polymorphism in historically understudied regions [32].

## Methods

### Samples used

Human cell line or patient-derived cellular RNA was collected from a variety of sources and in accordance with the University of Wisconsin and Dana Farber Cancer Research Center’s policy on human research subjects. Samples were anonymized prior to typing, and no genotyping results were released to physicians or used to inform clinical treatments. Samples were processed for RNA either as cell pellets, cell-culture homogenates, peripheral blood mononuclear cells, or whole blood. Samples included 15 patient samples from an unrelated HIV study at the University of Wisconsin, 40 HLA reference cell lines provided by the International Histocompatibility Working Group, 5 human embryonic cell-lines provided by WiCell, 40 samples derived from cell-lines or patients collected by the Dana Farber Cancer Center, and 10 blood samples from female Korean patients with HPV-16-induced cervical cancers from an unrelated study [33].

### RNA isolation and cDNA synthesis

Methods for isolation of RNA and cDNA synthesis were identical to those presented previously [24], involving the MagaNA Pure LC RNA isolation platform (Roche Applied Science, Indianapolis, IN), quantification using a NanoDrop 1000 spectrometer (ThermoFisher, Waltham, MA) and oligo(dT)_20_-primed SuperScript III cDNA synthesis (Invitrogen, Carlsbad, CA). For all experiments an input of 50 ng RNA was used to seed the cDNA synthesis.

### Universal primer design

The design of the 581 bp internally diagnostic amplicon has been previously described [24]. The method for finding additional conserved sites for HLA class I and HLA DRB universal primers was similar, and is briefly described here. All known HLA class I and DRB reference sequences from the IMGT/HLA database were aligned using MUSCLE [34] or CodonCode Aligner (CodonCode Corporation, Dedham, MA). All analysis was done with IMHT/HLA Release 3.4 (April 2011) [6]. In addition to the previously described HLA class I 581 bp amplicon spanning consensus positions 145–725 [24] we identified novel conserved primer binding sites in exons 1 and 7. Due to current read length limitations of GS-FLX Titanium reagents we could not fully sequence this amplicon. However, by combining this 1020 bp amplicon with the 581 bp amplicon acting as a bridge, we were able to tile nucleotides 50–1069 of the HLA class I open reading frame, including all of exons 2 through 6. To further normalize the distribution of reads, we altered these original 581 and 1 kb amplicons by mixing their forward and reverse primers. The class II DRB and other loci genotyping amplicon was identified with similar methods.

Initial sequencing with the HLA-1 forward primer revealed a sequence incompatibility with most HLA-A alleles, leading to the generation of a short, chimeric DNA bead which interfered with genotyping Additional file 4: Figure S1. To correct this issue, we switched the Roche/454 adapter positions, using adapter “B” with the forward gene-specific primer, and adapter “A” with the gene-specific reverse primer.

### Amplicon and library preparation

Fusion primers with Roche/454 adapters, MIDs, and gene-specific sequences were generated [Additional file 1: Table S3] and used to amplify PCR products using Phusion High-Fidelity DNA polymerase (New England Biolabs, Ipswich, MA) as previously described [24]. PCR products were purified with SPRI Ampure-XP paramagnetic beads (Beckman Coulter Genomics, Danvers, MA). DNA concentrations were normalized, and products were pooled into a single amplicon library. Due to the different sizes of our pooled amplicons, and the expected number of alleles sequenced, the three amplicons (HLA-1, HLA-2 and DRB amplicon) were pooled at a 3:6:1 ratio.

### Roche/454 Pyrosequencing

Emulsion PCR and pyrosequencing were performed with the amplicon (Lib-A) kit with GS Junior reagents from Roche/454 using standard, manufacturer provided protocols (454 Life Sciences, Branford, CT). An input DNA molecule-to-bead ratio of 1.5 was used. DNA bead enrichment levels were expected (between 5 and 20%) for all experiments, and sequencing yielded >70,000 filterpass reads per GS Junior run.

### Novel allele Sanger sequencing

One novel allele identified in sample KTS02 was confirmed through the use of Sanger sequencing. Primers specific to the novel allele (and not to the other alleles present in this individual) were used to amplify a primary PCR product from previously generated cDNA using High Fidelity Platinum Taq (Invitrogen, Carlsbad, CA). Products were purified with SPRI Ampure-XP paramagnetic beads, and sequenced on an Applied Biosystems 3730xl capillary sequencer (Applied Biosystems, Carlsbad, CA) using six different sequencing primers. Data was analyzed, and the novel SNP confirmed using CodonCode Aligner (CodonCode Corporation, Dedham, MA).

### Alignment algorithm, matrix creation and Galaxy implementation

After standard read filtering using the Roche/454 RunProcessor software, the primary sff file was parsed by MID and amplicon sequence using the sfffile utility (454 Life Sciences, Branford, CT). Individual sff files were converted into fastq files using BioPerl, and then submitted to a local Galaxy server for further analysis using the Galaxy API to write a data library and recursively execute mapping workflows using Perl and Python. Reads were quality- and length-filtered to a standardized length (between 300 and 350 bp depending on amplicon/read direction) and then mapped to the set of HLA reference alleles using BLAT [30]. Perfectly matching sequences were grouped, and matched alleles from each read direction were joined as described in the Results section. Positive allele rows were exported using a custom Galaxy tool, and the entire specific Galaxy workflow used is provided Additional file 2: File 1. The resulting matrix of allele matches was compared to expected match patterns to establish genotype. In some cases of low read numbers, potential homozygous loci, or novel alleles, manual interrogation of individual reads was necessary. RNA samples with poor amplifications or aberrantly sequenced reads were excluded from analysis.

## Abbreviations

HLA: Human Leukocyte Antigen; BLAT: BLAST-like Alignment Tool; SBT: Sequence-Based Typing; cSBT: cDNA Sequence-Based Typing; cDNA: complementary DNA; MID: Multiplex IDentifier; IMGT: International ImMunoGeneTics information system; PCR: Polymerase Chain Reaction; SSP: Sequence-Specific Primer assay; SSO: Sequence-Specific Oligonucleotide assay; SNP: Single Nucleotide Polymorphism; SPRI: Solid Phase Reversible Immobilization; emPCR: emulsion PCR; HIV: Human Immunodeficiency Virus.

## Competing interests

The authors declare that they have no competing interests.

## Authors’ contributions

SL designed the assay, experiments and analysis pipeline; participated in assay experiments; analyzed the data; and drafted the manuscript. BG carried out the assay experiments and assisted in data analysis. HC carried out confirmatory sequencing experiments. RW participated in experimental design troubleshooting and manuscript preparation. DK, VB,and ER provided samples and externally validated genotyping results. DO conceived of the study, participated in its design, helped draft the manuscript, and oversaw the project. All authors read and approved the final manuscript.

## Supplementary Material

Additional file 1: Table S1 In silico model of typing resolution for standard SBT genotyping assays which assess exons 2,3, exons 2,3,4 (class I) or exon 2 (DRB). **Table S2** - Primer sequences used for additional HLA class II loci typing. **Table S3** - Primers used. Table S4 - Read numbers recovered per sample and amplicon in both genotyping experiments. **Table S5** - Read numbers recovered per sample and amplicon in preliminary class II additional loci experiment. **Table S6** - Class I genotyping results from the second experiment. **Table S7** - DRB genotyping results from the second experiment. **Table S8** - Additional class II amplicon genotyping results. **Table S9** - Primers used for Sanger sequencing of novel KTS02 HLA-C allele. **Table S10** - Cost estimate for cSBT at a variety of multiplexing levels. Click here for file

Additional file 2: File 1 Galaxy workflow and custom tools/scripts used (zip archive). Click here for file

Additional file 3: File 2 Novel sequences identified during sequencing (fasta file). Click here for file

Additional file 4: Figure 1 Formation of abortive, short DNA products during emPCR. Click here for file
