# Supplementary figures and images for: Ultra-high resolution HLA genotyping and allele discovery by highly multiplexed cDNA amplicon pyrosequencing
Source: BMC Genomics. 2012 Aug 6;13:378. doi: 10.1186/1471-2164-13-378 (PMC3575390; doi:10.1186/1471-2164-13-378)

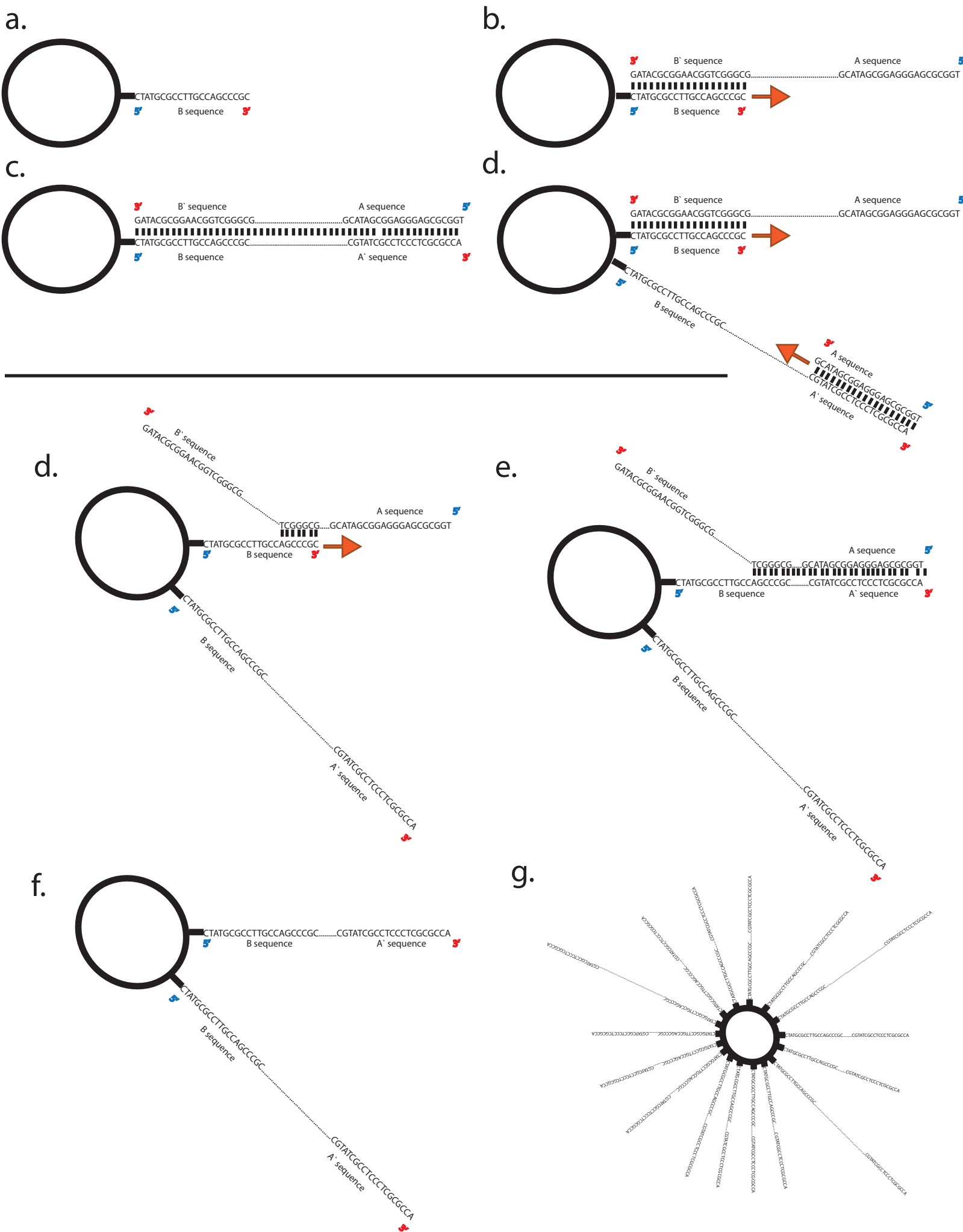

Supplement: Additional file 4: Figure 1 — Formation of abortive, short DNA products during emPCR. [file 1471-2164-13-378-S4.pdf]
